# A cross-sectional study on the associations between sugar- sweetened beverage consumption, a body shape index, and psychological symptoms among rural Chinese youth

**DOI:** 10.3389/fnut.2026.1913391

**Published:** 2026-10-26

**Authors:** Chao Gong Zeng, Chengjun Chai, JiaLi Xu, Mian Sha, Jian Xin

**Affiliations:** 1 Physical Education College of Shanghai University, Shanghai, China; 2 Physical Education College of Shangrao Normal University, Shangrao, China; 3 School of Physical Education, Chizhou University, Chizhou, China; 4 Party Committee Office of Gannan Medical University, Gannan, China

**Keywords:** a body shape index, adolescents, psychological symptoms, rural, sugar-sweetened beverage consumption

## Abstract

**Background:**

The rising prevalence of psychological symptoms among adolescents has become a serious public health concern worldwide. Nevertheless, previous studies have largely focused on urban or general populations, leaving a notable gap in research on rural adolescents – particularly regarding the associations between sugar-sweetened beverage (SSB) consumption, a body shape index (ABSI, a measure of body composition), and psychological symptoms. This study aims to fill that gap by providing empirical evidence from rural China, thereby offering a locally grounded reference for developing early intervention and targeted prevention strategies.

**Methods:**

In this study, a stratified cluster sampling design was adopted to carry out a cross-sectional evaluation of SSB consumption, ABSI, and psychological symptoms among 52,735 rural adolescents aged 12–18 years, of whom 26,043 were boys. To examine the associations between these variables, we applied a range of statistical tests, including *t*-tests, Chi-square tests, one-way ANOVA, binary logistic regression, and binary logistic regression embedded within generalized linear models.

**Results:**

The overall prevalence of psychological symptoms among rural Chinese adolescents was 21.8%, with a significantly higher rate in males (22.5%) than in females (21.2%; χ^2^ = 13.126, *P* < 0.001). Weekly SSB consumption exceeding five times was reported by 14.5% of the participants, and the mean ABSI score was 0.06 ± 0.01. After adjustment for relevant covariates, adolescents who consumed SSB > 5 times/week and fell within the 51st–75th percentile range of ABSI exhibited the highest odds of psychological symptoms (OR = 2.40, 95% CI: 2.10–2.73; *P* < 0.001), indicating more than twofold greater odds of psychological symptoms.

**Conclusion:**

Both independent and combined associations were observed between SSB consumption, ABSI, and psychological symptoms among rural Chinese adolescents. Future interventions targeting psychological symptoms may consider SSB consumption and ABSI factors to better inform intervention strategies; however, these cross-sectional associations do not imply causation.

## 1 Background

In recent years, lifestyle transformations have been accompanied by reduced physical activity and increased sedentary time, contributing to a sustained rise in the prevalence of adolescent psychological symptoms. This upward trend adversely affects both academic performance and long-term career outcomes, and has consequently emerged as a global public health concern (1, 2). In a meta-analysis encompassing more than 1.38 million participants, the pooled prevalence of adolescent psychological symptoms was estimated at 30%, with a clear upward trend over time (3). Meanwhile, the prevalence of psychological symptoms among adolescents in countries such as the United States and Europe ranges from 30% to 60%, and is also on the rise (4, 5). This trend, however, is no longer confined to developed nations but is progressively extending to developing regions. In Africa, survey data similarly indicate a steady rise in the prevalence of psychological symptoms among adolescents, which poses a significant threat to their healthy development (3). China, as a developing country, is similarly affected; national surveys report a prevalence of 31% among its adolescent population, with a sustained upward trajectory, highlighting a grave challenge for mental health prevention and control efforts (6). In recent years, however, the increasing prevalence of adolescent psychological symptoms has been expanding beyond China’s urban centres into economically disadvantaged rural areas, thereby constituting a significant risk to the mental well-being of young people in these regions (7). A longitudinal study revealed that from 2018 to 2022, the prevalence of psychological symptoms among adolescents in rural China (35.10%) exceeded that of their urban counterparts (30.16%), with a faster rate of increase warranting attention and concern (8). Past research has also shown that factors influencing adolescents’ psychological symptoms are multifaceted, encompassing various aspects such as eating behaviors, sleep patterns, body composition, and physical activity (9, 10). However, previous studies have paid little attention to SSB consumption among adolescents in rural China and the novel indicator ABSI reflecting body composition, with even fewer investigations into their association with psychological symptoms.

SSB consumption is associated with excessive intake of free sugars and may contribute to a range of issues including obesity and endocrine disorders. This phenomenon has been suggested to exert adverse effects on the physical and mental well-being of adolescents, and has consequently emerged as one of the foremost public health challenges confronting nations globally (11, 12). A study compiling extensive data from 1990 to 2020 at global, regional, and national levels reveals that in 2020, the global average weekly consumption of sugar-sweetened beverages (SSBs) was 2.6 cups (8 ounces per cup). Latin America and the Caribbean had the highest consumption (7.3 cups per week), while South Asia had the lowest (0.7 cups per week). Urban areas also showed higher consumption than rural areas (13). Pooling data from 15 cohorts and more than 1.21 million individuals, a meta-analysis found that those with high SSB intake had a 12% greater risk of dying from any cause (14). An umbrella review synthesizing 40 prospective cohort studies further corroborated the positive association between SSB consumption and all-cause mortality, with a 4% increase in mortality risk for each additional 250 mL/day of intake (15). Additionally, SSB consumption has been implicated in the development of chronic cardiovascular diseases, diabetes, and other health conditions (16). However, it is noteworthy that excessive SSB consumption is spreading from developed to developing countries, posing a threat to the health of adolescents in developing nations (17). For example, one study projects that global SSB consumption will increase by approximately 9.54% by 2050, with South Asia expected to see the largest growth. This research indicates that the center of gravity for SSB consumption is shifting toward developing countries (18). Similarly, China is no exception. A study synthesizing the burden of deaths and diseases attributable to excessive consumption of sugar-sweetened beverages (SSBs) among the Chinese population from 1990 to 2019 indicates that in 2019, deaths due to excessive SSB intake reached 46,000 in China – a 95% increase compared to 1990. This finding indicates that over the past three decades, the mortality risk associated with excessive consumption of sugar-sweetened beverages has significantly increased for the Chinese population (19). Research also indicates that excessive SSB consumption can lead to endocrine disorders and neurological impairments, adversely affecting the development of mental health (20). In a study of U.S. adults, consuming sugar-sweetened beverages at least once per day was associated with a 26% higher prevalence of reporting poor mental health for 14 or more days during the preceding month (21). Given that adolescent SSB consumption is now extending from urban centers into the vast rural countryside, it becomes imperative to investigate its association with the prevalence of psychological symptoms among rural youth. Regrettably, previous research examining this specific relationship within China’s rural adolescent population remains scarce.

Body composition exhibits a strong association with psychological symptoms. Research indicates that an increase in BMI, a measure of body composition, has been positively associated with a higher prevalence of psychological symptoms. Obesity is associated with an elevated risk of developing depression (OR = 1.17–1.68) and anxiety (OR = 1.25–1.71) (22). In recent years, with increasing attention to adolescents’ body composition, research on multiple indicators reflecting body composition has grown substantially. Studies indicate that indicators such as BMI, BRI, waist circumference, and waist-to-height ratio – all reflecting body composition – exhibit significant associations with psychological symptoms (23). However, in recent years, as research has deepened, studies examining the relationship between the ABSI index – which reflects body composition – and health have proliferated. Unlike traditional BMI, ABSI is a comprehensive indicator of body composition derived from the combined calculation of three dimensions: height, weight, and waist circumference. This approach better reflects the body’s visceral fat content (24). Research indicates that ABSI provides a more accurate reflection of body composition compared to indicators such as BMI and waist circumference, and demonstrates superior predictive value for chronic disease onset (25). Another study also demonstrated that compared to BMI, ABSI is significantly more effective in predicting all-cause mortality risk, exhibiting higher sensitivity (26). To date, research on ABSI has largely concentrated on its associations with chronic diseases, whereas investigations into its relationship with psychological symptoms remain sparse. Given the ongoing development of rural China, adolescent obesity in these areas is an issue that increasingly demands attention. Consequently, exploring the link between ABSI and psychological symptoms could serve as an effective strategy for preventing the onset of such symptoms.

However, other studies have found that factors influencing adolescents‘ psychological symptoms are multifaceted, with the focus being influenced by dietary behaviors and body composition (27, 28). Adolescents in rural China constitute a significant proportion of the nation’s youth population, making their health status worthy of attention and concern. Unfortunately, past research has predominantly focused on the individual associations between body composition or dietary behaviors and psychological symptoms, with limited studies examining the combined effects of these factors on psychological symptoms. Furthermore, previous investigations have primarily targeted urban adolescent populations, while research on rural adolescents remains relatively scarce. Accordingly, this cross-sectional survey was conducted among adolescents in rural China to investigate both the independent and combined associations of SSB consumption and ABSI with psychological symptoms. Of note, this combined-category analysis describes the joint distribution of the two exposures and does not constitute a formal statistical test of interaction. The findings are intended to serve as a reference and to offer valuable insights for the prevention and intervention of psychological symptoms in this rural adolescent population.

## 2 Methods

### 2.1 Study design and participants

A multistage stratified cluster sampling design was adopted to recruit the study participants. First, based on China’s geographical distribution and the principle of feasibility, five provincial-level administrative regions – Guangdong (south), Heilongjiang (northeast; the survey was conducted in Harbin), Xinjiang (northwest), Anhui (east), and Henan (central China) – were purposively selected to represent the country’s five major geographic areas. Second, six rural areas were selected as test sites within each region. Third, two complete secondary schools were selected as test schools within each rural area. The survey was conducted during the 2024–2025 academic year. In accordance with testing requirements, eligible junior high and senior high school students within the survey areas were included. The study assessed 53,041 students aged 12–18 across 60 rural schools. Following the assessment, 306 ineligible students were excluded, yielding 52,735 valid responses with a questionnaire validity rate of 99.42% (52,735 of 53,041 assessed); all assessments were conducted during scheduled school hours, and students who were absent on the survey day were not included in the 53,041 assessed, so this figure reflects the proportion of valid questionnaires among assessed students rather than a conventional response rate. The sample comprised 26,043 boys and 26,692 girls. Inclusion criteria were: enrolled junior and senior high school students whose parents and the student themselves held rural household registration, and whose parents/guardians and the student voluntarily agreed to participate in the assessment and survey. Exclusion criteria were: damaged questionnaires, missing key demographic information, or refusal by parents or the student to participate. The sampling process for participants at each stage is illustrated in Figure 1.

**FIGURE 1:**
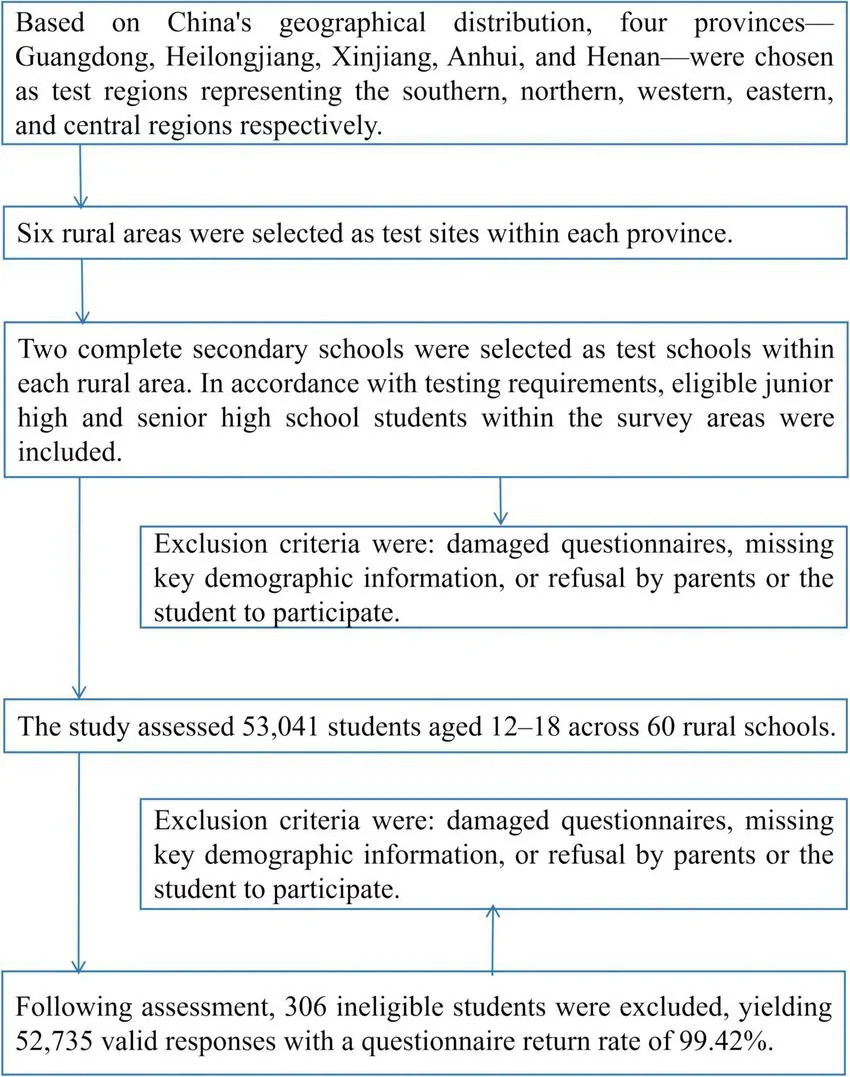
Sampling procedure for adolescent participants aged 12–18 in rural China.

We conducted this study in line with the Declaration of Helsinki. Parents or guardians gave their written assent, and the participating adolescents also signed their own consent forms. For participants younger than 16, we obtained informed consent from their parents or legal guardians separately. Ethical clearance was granted by the Biomedical Research Ethics Committee of Gannan Medical University (No. 2024724).

### 2.2 Assessment of exposure

#### 2.2.1 SSB consumption

In this study, the consumption of SSBs was evaluated using a self-administered Food Frequency Questionnaire (FFQ) (29). This questionnaire includes specific items regarding SSB consumption, covering the frequency of drinking both sugar-sweetened beverages and sugar-free soft drinks. Each item comprises the following response options: “Never or less than once a month”; “1–3 times a month”; “Once a week”; “2–4 times a week”; “5–6 times a week”; “1 time per day”; “2–3 times per day”; “4–5 times per day”; “6 or more times per day.” For analytical convenience and practicality in this study, SSB consumption was categorized into three groups: < 2 times/week, 3–5 times/week, and > 5 times/week. This questionnaire has been validated in multiple studies and demonstrates good reliability and validity(30–32). Furthermore, this questionnaire has previously been applied to Chinese adolescent populations and has been shown to possess sound reliability and validity(33).

#### 2.2.2 A body shape index (ABSI)

ABSI is an index for assessing body shape, body composition, and health risks. Proposed by Nir Krakauer in 2012, it aims to address the limitations of traditional BMI, which cannot distinguish body fat distribution (34). The calculation formula is: ABSI = Waist Circumference (m)/(BMI^∧^(2/3) × Height^∧^(1/2)). In this formula, waist circumference reflects abdominal fat accumulation, BMI assesses overall obesity through the relationship between weight and height, and height is used to standardize body proportions. To assess ABSI, participants’ waist circumference, weight, and height are first measured. After calculating BMI, the ABSI value is derived by substituting into the formula. In this study, measurements of height, weight, and waist circumference were conducted using instruments and evaluation protocols that met the standards stipulated by the National Student Physical Fitness Survey. ABSI’s advantage lies in its ability to predict mortality risk independently of BMI, demonstrating particular sensitivity for “normal-weight obesity” individuals (those with normal BMI but excessive waist circumference).

### 2.3 Ascertainment of outcome

The outcome variable of this study is psychological symptoms. The Brief Adolescent Mental Health Screening Questionnaire was administered to assess psychological symptoms among adolescents(35, 36). This questionnaire consists of 15 items, each offering 6 response options: “Lasted over 3 months,” “Lasted over 2 months,” “Lasted over 1 month,” “Lasted over 2 weeks,” “Lasted over 1 week,” and “Did not occur or lasted less than 1 week.” Participants should select the appropriate option based on their actual experiences over the past 6 months. Participants scoring 1, 2, or 3 items receive 1 point, while those scoring 4, 5, or 6 items receive 0 points. The total score is the sum of points across all 15 items. The scale comprises three dimensions: Emotional problems (items 1–7), Behavioral problems (items 8, 10, 11, 13), and Social adjustment difficulties (items 9, 12, 14, 15). Social adjustment difficulties comprise 4 items: 9, 12, 14, and 15. Cutoffs for positive results were set at ≥ 4 for Emotional problems, ≥ 1 for Behavioral problems, and ≥ 2 for Social adjustment difficulties, with a total score ≥ 7 signaling psychological symptoms. These cut-offs have been adopted and validated in previous studies of Chinese adolescents (35, 36). The questionnaire shows good reliability and validity among Chinese adolescents and has been widely used in research (35, 36).

### 2.4 Assessment of covariates

Based on prior work and our own results, the covariates we adjusted for were standing long jump, parents’ education, commuting method, snacks, and sleep time. Parental education was divided into four levels: ≤ primary, middle school, high school, and ≥ college. Ways of going to and from school are categorized into positive ways and negative ways. Positive ways include walking and cycling. Negative ways include taking buses, subways, private cars, and riding electric scooters. Snack food is divided into three categories: < 2 times/week, 3–5 times/week, and > 5 times/week. Sleep duration is calculated based on the time the participant falls asleep and wakes up. This study divided sleep duration into three categories: < 6 h/day, 6–8 h/day, and ≥ 8 h/day.

### 2.5 Statistical analyses

The results are presented with both continuous and categorical variables. Continuous data are reported as mean ± standard deviation, whereas categorical data are expressed as percentages. Between-sex differences in continuous variables were examined using *t*-tests, while comparisons of psychological symptom prevalence and other categorical outcomes were performed using Chi-square tests.

In the present study, ABSI was stratified by age and sex and then divided into quartiles, yielding four groups: < 25th, 25th–50th, 51st–75th, and > 75th percentiles. The associations of SSB consumption and ABSI with psychological symptoms were examined using Chi-square tests, binary logistic regression, and binary logistic regression embedded in generalized linear models. Binary logistic regressions were performed with the presence of psychological symptoms as the dependent variable and SSB intake and ABSI quartiles as independent variables. Three sequential models were constructed: Model 1 was unadjusted (crude); Model 2 additionally controlled for age, paternal and maternal education, and commuting mode to and from school; and Model 3 further adjusted for standing long jump performance, snack consumption, and sleep duration on top of Model 2. To examine the combined associations of SSB consumption and ABSI categories with psychological symptoms, we employed a generalized-linear-model-based binary logistic regression, with the same dependent variable and both exposures as independent variables, while adjusting for all the aforementioned covariates (age, parental education, commute mode, standing long jump, snacks, and sleep). Of note, this combined-category analysis describes the joint distribution of the two exposures across strata; it does not constitute a formal statistical test of interaction (i.e., no multiplicative interaction term was added to the model), and thus should not be interpreted as evidence of synergy or antagonism. All results are reported as odds ratios (ORs) with 95% confidence intervals.

## 3 Results

We evaluated 52,735 rural Chinese adolescents (26,043 boys, 26,692 girls) for SSB intake, ABSI, and psychological symptoms. Their average age was 15.09 ± 1.89 years. The prevalence of psychological symptoms stood at 21.8%, significantly higher among boys (22.5%) than girls (21.2%, χ^2^ = 13.126, *P* < 0.001). SSB consumption patterns were: 33.1% (<2/week), 52.4% (3–5/week), and 14.5% (>5/week). The overall ABSI was 0.06 ± 0.01. Table 1 summarizes sex-specific baseline characteristics.

**TABLE 1:** Comparative analysis of basic characteristics of adolescents in rural China by sex.

Classification	Boys	Girls	Total	χ ^2^ /*t*-value	*P*-value
Number	26043	26692	52735		
Age	15.03 ± 1.87	15.16 ± 1.9	15.09 ± 1.89	−7.797	<0.001
Height	169.83 ± 9.15	161.11 ± 6.4	165.41 ± 9	127.079	<0.001
Weight	59.56 ± 13.42	51.5 ± 9.16	55.48 ± 12.15	80.783	<0.001
Body mass index(BMI)	20.51 ± 3.69	19.8 ± 3.13	20.15 ± 3.44	23.808	<0.001
Waist circumference	72.13 ± 11.31	66.41 ± 9.01	69.23 ± 10.60	64.339	<0.001
Abody shape index (ABSI)	0.06 ± 0.01	0.06 ± 0.01	0.06 ± 0.01	−2.000	0.045
Standing long jump	207.51 ± 32.08	168.28 ± 21.39	187.65 ± 33.53	165.61	<0.001
Father’s educational attainment				5.91	0.116
≤Primary	2990(11.5)	3126(11.7)	6116(11.6)		
Middle school	9322(35.8)	9356(35.1)	18678(35.4)		
High school	8556(32.9)	8987(33.7)	17543(33.3)		
≥College	5175(19.9)	5223(19.6)	10398(19.7)		
Mother’s educational attainment				6.856	0.077
≤Primary	4659(17.9)	4697(17.6)	9356(17.7)		
Middle school	8833(33.9)	9225(34.6)	18058(34.2)		
High school	7928(30.4)	8226(30.8)	16154(30.6)		
≥College	4623(17.8)	4544(17)	9167(17.4)		
Ways of going to and from school				130.609	<0.001
Positive way	12656(48.6)	11647(43.6)	24303(46.1)		
Negative way	13387(51.4)	15045(56.4)	28432(53.9)		
Snack food				757.612	<0.001
<2 times/week	5865(22.5)	7989(29.9)	13854(26.3)		
3–5 times/week	14643(56.2)	15148(56.8)	29791(56.5)		
>5 times/week	5535(21.3)	3555(13.3)	9090(17.2)		
Sleep duration				124.364	<0.001
<6 h/day	4061(15.6)	4499(16.9)	8560(16.2)		
6–8 h/day	18004(69.1)	18990(71.1)	36994(70.2)		
≥8 h/day	3978(15.3)	3203(12)	7181(13.6)		
ABSI quartile				125.517	<0.001
<25th	7048(27.1)	6296(23.6)	13344(25.3)		
25–50th	6746(25.9)	7141(26.8)	13887(26.3)		
51–75th	5930(22.8)	6944(26)	12874(24.4)		
>75th	6319(24.3)	6311(23.6)	12630(23.9)		
SSB consumption				643.736	<0.001
<2 times/week	7473(28.7)	9984(37.4)	17457(33.1)		
3–5 times/week	14000(53.8)	13616(51)	27616(52.4)		
>5 times/week	4570(17.5)	3092(11.6)	7662(14.5)		
Emotional problems [N(%)]	7057(27.1)	7136(26.7)	14193(26.9)	0.882	0.348
Behavioral problems [N(%)]	7092(27.2)	6520(24.4)	13612(25.8)	54.164	<0.001
Social adjustment difficulties [N(%)]	4846(18.6)	4523(16.9)	9369(17.8)	24.938	<0.001
Psychological symptoms [N (%)]	5851(22.5)	5649(21.2)	11500(21.8)	13.126	<0.001

ABSI, A Body Shape Index; CI, confidence interval; OR, odds ratio; SSB, sugar-sweetened beverage consumption.

Univariate analysis of psychological symptom prevalence among adolescents in rural China revealed statistically significant differences across the following factors: sex, father’s educational attainment, mother’s educational attainment, commuting methods, snack food consumption, sleep duration, ABSI quartile, and SSB consumption (χ^2^ = 13.126, 70.366, 77.731, 25.308, 217.312, 848.78, 71.824, 83.149, *P* < 0.001). Detailed results are presented in Table 2.

**TABLE 2:** Comparative analysis of demographic characteristics, SSB consumption, ABSI, and psychological symptoms among adolescents in rural China.

Classification	Psychological symptoms [N(%)]		χ ^2^ /*t*-value	*P*-value
	No	Yes		
Number	41235	11500		
Sex			13.126	<0.001
Boys	20192(77.5)	5851(22.5)		
Girls	21043(78.8)	5649(21.2)		
Father’s educational attainment			70.366	<0.001
Primary school or below	4570(74.7)	1546(25.3)		
Middle School	14491(77.6)	4187(22.4)		
High School	13893(79.2)	3650(20.8)		
College degree or above	8281(79.6)	2117(20.4)		
Mother’s educational attainment			77.731	<0.001
Primary school or below	7075(75.6)	2281(24.4)		
Middle School	14037(77.7)	4021(22.3)		
High School	12716(78.7)	3438(21.3)		
College degree or above	7407(80.8)	1760(19.2)		
Ways of going to and from school			25.308	<0.001
Positive way	19241(79.2)	5062(20.8)		
Negative way	21994(77.4)	6438(22.6)		
Snack food			217.312	<0.001
<2 times/week	10222(73.8)	3632(26.2)		
3–5 times/week	23823(80)	5968(20)		
>5 times/week	7190(79.1)	1900(20.9)		
Sleep duration			848.78	<0.001
<6 h/day	5685(66.4)	2875(33.6)		
6–8 h/day	29638(80.1)	7356(19.9)		
≥8 h/day	5912(82.3)	1269(17.7)		
ABSI quartile			71.824	<0.001
<25th	10687(80.1)	2657(19.9)		
25–50th	10975(79)	2912(21)		
51–75th	9965(77.4)	2909(22.6)		
>75th	9608(76.1)	3022(23.9)		
SSB consumption			83.149	<0.001
<2 times/week	13874(79.5)	3583(20.5)		
3–5 times/week	21662(78.4)	5954(21.6)		
>5 times/week	5699(74.4)	1963(25.6)		

ABSI, A Body Shape Index; CI, confidence interval; OR, odds ratio; SSB, sugar-sweetened beverage consumption.

Table 3 presents univariate comparisons of SSB, ABSI, and psychological symptoms among adolescents in rural China. Significant sex differences were observed in the prevalence of behavioral problems, social adjustment difficulties, and overall psychological symptoms (all *P* < 0.001), whereas the sex difference in emotional problems was not statistically significant (*P* = 0.348; Table 1).

**TABLE 3:** Univariate associations of SSB consumption, ABSI, and psychological symptoms among rural Chinese adolescents.

Classification	N	Emotional problems			Behavioral problems			Social adjustment difficulties			Psychological symptoms		
		N (%)	χ ^2^-value	*P-*value	N (%)	χ ^2^-value	*P*-value	N (%)	χ ^2^-value	*P*-value	N (%)	χ ^2^-value	*P*-value
Boys													
ABSI quartile			38.662	<0.001		34.835	<0.001		32.356	<0.001		52.107	<0.001
<25th		1772 (25.1)			1833 (26)			1213 (17.2)			1416 (20.1)		
25–50th		1769 (26.2)			1747 (25.9)			1176 (17.4)			1455 (21.6)		
51–75th		1640 (27.7)			1620 (27.3)			1196 (20.2)			1412 (23.8)		
>75th		1876 (29.7)			1892 (29.9)			1261 (20)			1568 (24.8)		
SSB		1987 (26.6)	38.186	<0.001	1997 (26.7)	25.789	<0.001	1363 (18.2)	29.863	<0.001	1586 (21.2)	29.984	<0.001
<2													
3-5		3664 (26.2)			3712 (26.5)			2503 (17.9)			3104 (22.2)		
>5		1406 (30.8)			1383 (30.3)			980 (21.4)			1161 (25.4)		
Girls													
ABSI quartile		1670 (26.5)	24.804	<0.001	1493 (23.7)	33.989	<0.001	959 (15.2)	27.397	<0.001	1241 (19.7)	24.391	<0.001
<25th													
25–50th		1811 (25.4)			1662 (23.3)			1190 (16.7)			1457 (20.4)		
51–75th		1823 (26.3)			1651 (23.8)			1195 (17.2)			1497 (21.6)		
>75th		1832 (29)			1714 (27.2)			1179 (18.7)			1454 (23)		
SSB		2543 (25.5)	55.029	<0.001	2329 (23.3)	67.85	<0.001	1640 (16.4)	73.864	<0.001	1997 (20)	50.757	<0.001
<2													
3–5		3599 (26.4)			3252 (23.9)			2191 (16.1)			2850 (20.9)		
>5		994 (32.1)			939 (30.4)			692 (22.4)			802 (25.9)		
Total													
ABSI quartile		3442 (25.8)	55.966	<0.001	3326 (24.9)	67.647	<0.001	2172 (16.3)	51.876	<0.001	2657 (19.9)	71.824	<0.001
<25th													
25–50th		3580 (25.8)			3409 (24.5)			2366 (17)			2912 (21)		
51–75th		3463 (26.9)			3271 (25.4)			2391 (18.6)			2909 (22.6)		
>75th		3708 (29.4)			3606 (28.6)			2440 (19.3)			3022 (23.9)		
SSB		4530 (25.9)	89.284	<0.001	4326 (24.8)	95.579	<0.001	3003 (17.2)	101.24	<0.001	3583 (20.5)	83.149	<0.001
<2													
3-5		7263 (26.3)			6964 (25.2)			4694 (17)			5954 (21.6)		
>5		2400 (31.3)			2322 (30.3)			1672 (21.8)			1963 (25.6)		

ABSI, A Body Shape Index; CI, confidence interval; OR, odds ratio; SSB, sugar-sweetened beverage consumption.

Binary logistic regression was performed to examine the associations of ABSI and SSB consumption with psychological symptoms among adolescents in rural China, with psychological symptoms set as the outcome variable and ABSI and SSB intake as the predictors. After adjusting for relevant covariates, the results showed that adolescents in the ABSI quartile > 75th had 1.23-fold higher odds of psychological symptoms compared with those in the ABSI quartile < 25th (*P* < 0.001). Similarly, adolescents with SSB consumption > 5 times/week had 1.21-fold higher odds of psychological symptoms compared with those with SSB consumption < 2 times/week (*P* < 0.001). The same trend was observed in analyses stratified by sex, with detailed results presented in Table 4.

**TABLE 4:** Binary logistic regression analysis of SSB intake, ABSI, and psychological symptoms in rural Chinese adolescents.

Sex/Variable	Group	Psychological symptoms					
		Model 1		Model 2		Model 3	
		OR (95% CI)	*P-*value	OR (95% CI)	*P-*value	OR (95% CI)	*P-*value
Boys							
ABSI quartile	<25th	1.00		1.00		1.00	
	25–50th	1.09 (1.01∼1.19)	0.033	1.09 (1.00∼1.18)	0.052	1.11 (1.02∼1.20)	0.017
	51–75th	1.24 (1.14∼1.35)	<0.001	1.22 (1.12∼1.32)	<0.001	1.25 (1.15∼1.36)	<0.001
	>75th	1.31 (1.21∼1.42)	<0.001	1.25 (1.15∼1.35)	<0.001	1.28 (1.18∼1.40)	<0.001
SSB	<2	1.00		1.00		1.00	
	3–5	1.06 (0.99∼1.13)	0.109	1.06 (0.99∼1.14)	0.085	1.02 (0.95∼1.10)	0.542
	>5	1.26 (1.16∼1.38)	<0.001	1.31 (1.20∼1.43)	<0.001	1.14 (1.03∼1.25)	0.008
Girls							
ABSI quartile	<25th	1.00		1.00		1.00	
	25–50th	1.04 (0.96∼1.14)	0.317	1.05 (0.97∼1.15)	0.230	1.08 (0.99∼1.18)	0.069
	51–75th	1.12 (1.03∼1.22)	0.009	1.11 (1.02∼1.21)	0.017	1.15 (1.06∼1.26)	0.001
	>75th	1.22 (1.12∼1.33)	<0.001	1.16 (1.07∼1.27)	0.001	1.20 (1.10∼1.31)	<0.001
SSB	<2	1.00		1.00		1.00	
	3–5	1.06 (0.99∼1.13)	0.081	1.05 (0.99∼1.12)	0.118	0.99 (0.92∼1.05)	0.673
	>5	1.40 (1.28∼1.54)	<0.001	1.44 (1.31∼1.58)	<0.001	1.23 (1.12∼1.36)	<0.001
Total							
ABSI quartile	<25th	1.00		1.00		1.00	
	25–50th	1.07 (1.01∼1.13)	0.031	1.07 (1.01∼1.13)	0.033	1.09 (1.02∼1.15)	0.007
	51–75th	1.17 (1.11∼1.25)	<0.001	1.16 (1.09∼1.23)	<0.001	1.18 (1.11∼1.26)	<0.001
	>75th	1.27 (1.19∼1.34)	<0.001	1.20 (1.13∼1.27)	<0.001	1.23 (1.16∼1.31)	<0.001
SSB	<2	1.00		1.00		1.00	
	3-5	1.06 (1.02∼1.12)	0.009	1.06 (1.02∼1.11)	0.010	1.02 (0.97∼1.07)	0.385
	>5	1.33 (1.25∼1.42)	<0.001	1.38 (1.29∼1.47)	<0.001	1.21 (1.13∼1.30)	<0.001

ABSI, A Body Shape Index; CI, confidence interval; OR, odds ratio; SSB, sugar-sweetened beverage consumption.

As shown in Figure 2, the odds of psychological symptoms increased with rising levels of SSB consumption and ABSI quartiles.

**FIGURE 2:**
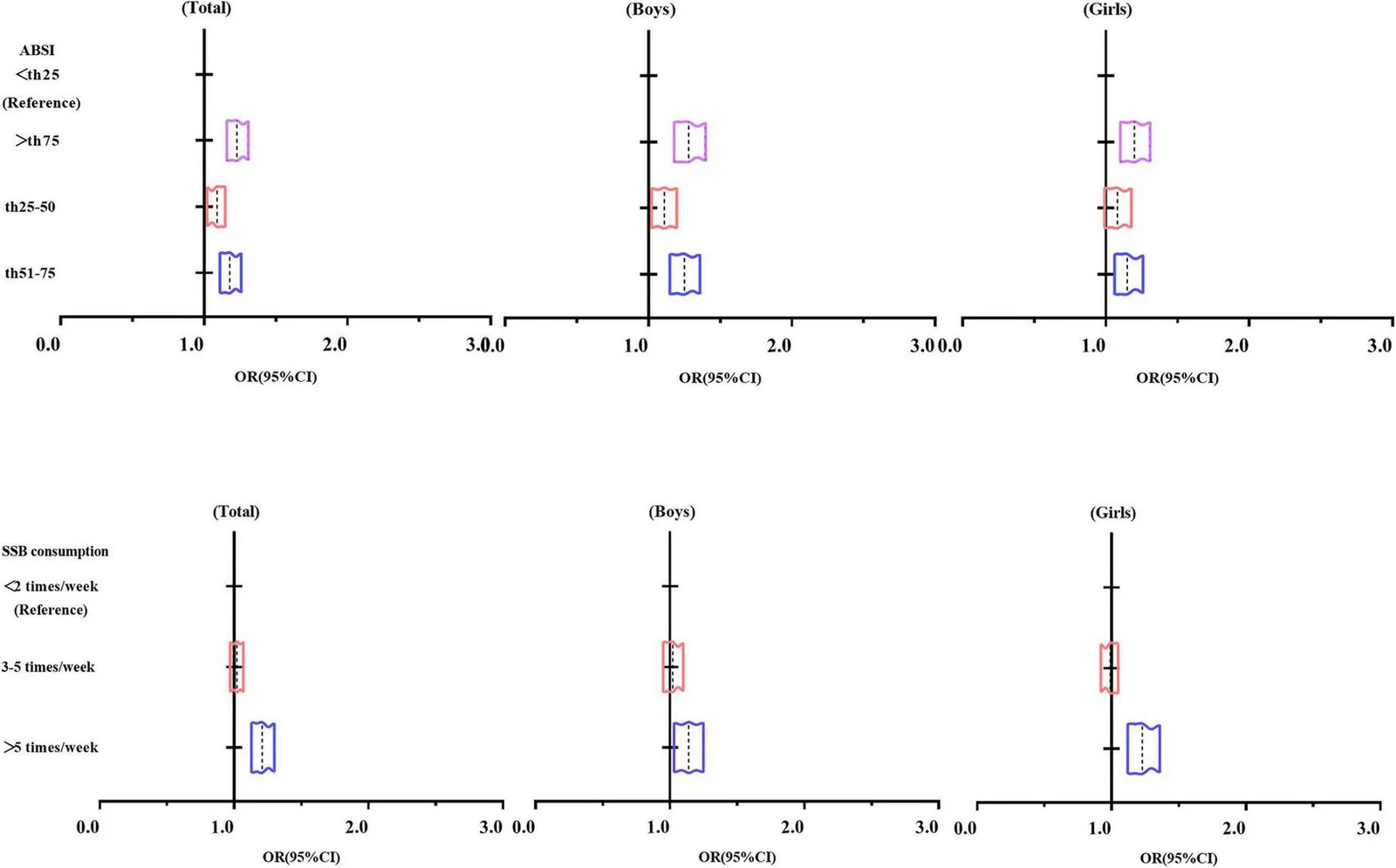
Trends in odds ratio values from binary logistic regression analysis of SSB consumption, ABSI, and psychological symptoms among adolescents in rural China.

A binary logistic regression analysis was performed within a generalized linear model framework, using the combined categories of ABSI and SSB consumption as independent variables and the presence of psychological symptoms as the dependent variable among rural Chinese adolescents. After adjustment for relevant covariates, those who consumed SSB more than five times per week and fell into the 51st–75th percentile range of ABSI were found to have the highest odds of psychological symptoms (OR = 2.40, 95% CI: 2.10–2.73, *P* < 0.001). This pattern remained consistent in sex–stratified analyses, the detailed results of which are presented in Table 5.

**TABLE 5:** Generalized linear model-based binary logistic regression analysis of SSB consumption, ABSI, and psychological symptoms among rural Chinese adolescents.

Sex	Classification of interaction		Psychological symptoms	
	SSB	ABSI quartile	*OR* (95% *CI*)	*P*-value
Boys	<2 times/week	<25th	1.00	
		25–50th	1.39 (1.18∼1.63)	<0.001
		51–75th	1.35 (1.14∼1.60)	<0.001
		>75th	1.95 (1.67∼2.29)	<0.001
	3–5 times/week	<25th	1.45 (1.26∼1.68)	<0.001
		25–50th	1.35 (1.17∼1.56)	<0.001
		51–75th	1.56 (1.35∼1.81)	<0.001
		>75th	1.59 (1.38∼1.84)	<0.001
	>5 times/week	<25th	1.39 (1.16∼1.66)	<0.001
		25–50th	1.77 (1.48∼2.11)	<0.001
		51–75th	2.42 (2.03∼2.90)	<0.001
		>75tht	1.73 (1.44∼2.07)	<0.001
Girls	<2 times/week	<25th	1.00	
		25–50th	1.32 (1.13∼1.53)	<0.001
		51–75th	1.39 (1.20∼1.62)	<0.001
		>75th	1.89 (1.64∼2.19)	<0.001
	3–5 times/week	<25th	1.55 (1.35∼1.78)	<0.001
		25–50th	1.40 (1.22∼1.61)	<0.001
		51–75th	1.47 (1.28∼1.68)	<0.001
		>75th	1.46 (1.27∼1.68)	<0.001
	>5 times/week	<25th	1.77 (1.45∼2.15)	<0.001
		25–50th	1.83 (1.52∼2.22)	<0.001
		51–75th	2.33 (1.92∼2.82)	<0.001
		>75th	1.87 (1.52∼2.30)	<0.001
Total	<2 times/week	<25th	1.00	
		25–50th	1.34 (1.20∼1.50)	<0.001
		51–75th	1.37 (1.23∼1.53)	<0.001
		>75th	1.92 (1.72∼2.14)	<0.001
	3–5 times/week	<25th	1.51 (1.36∼1.66)	<0.001
		25–50th	1.38 (1.25∼1.53)	<0.001
		51–75th	1.51 (1.37∼1.67)	<0.001
		>75th	1.53 (1.38∼1.69)	<0.001
	>5 times/week	<25th	1.54 (1.35∼1.76)	<0.001
		25–50th	1.81 (1.59∼2.06)	<0.001
		51–75th	2.40 (2.10∼2.73)	<0.001
		>75th	1.80 (1.57∼2.06)	<0.001

ABSI, A Body Shape Index; CI, confidence interval; OR, odds ratio; SSB, sugar-sweetened beverage consumption.

Figure 3 demonstrates that the overall odds of psychological symptoms among rural Chinese adolescents rose continuously as SSB consumption and ABSI increased.

**FIGURE 3:**
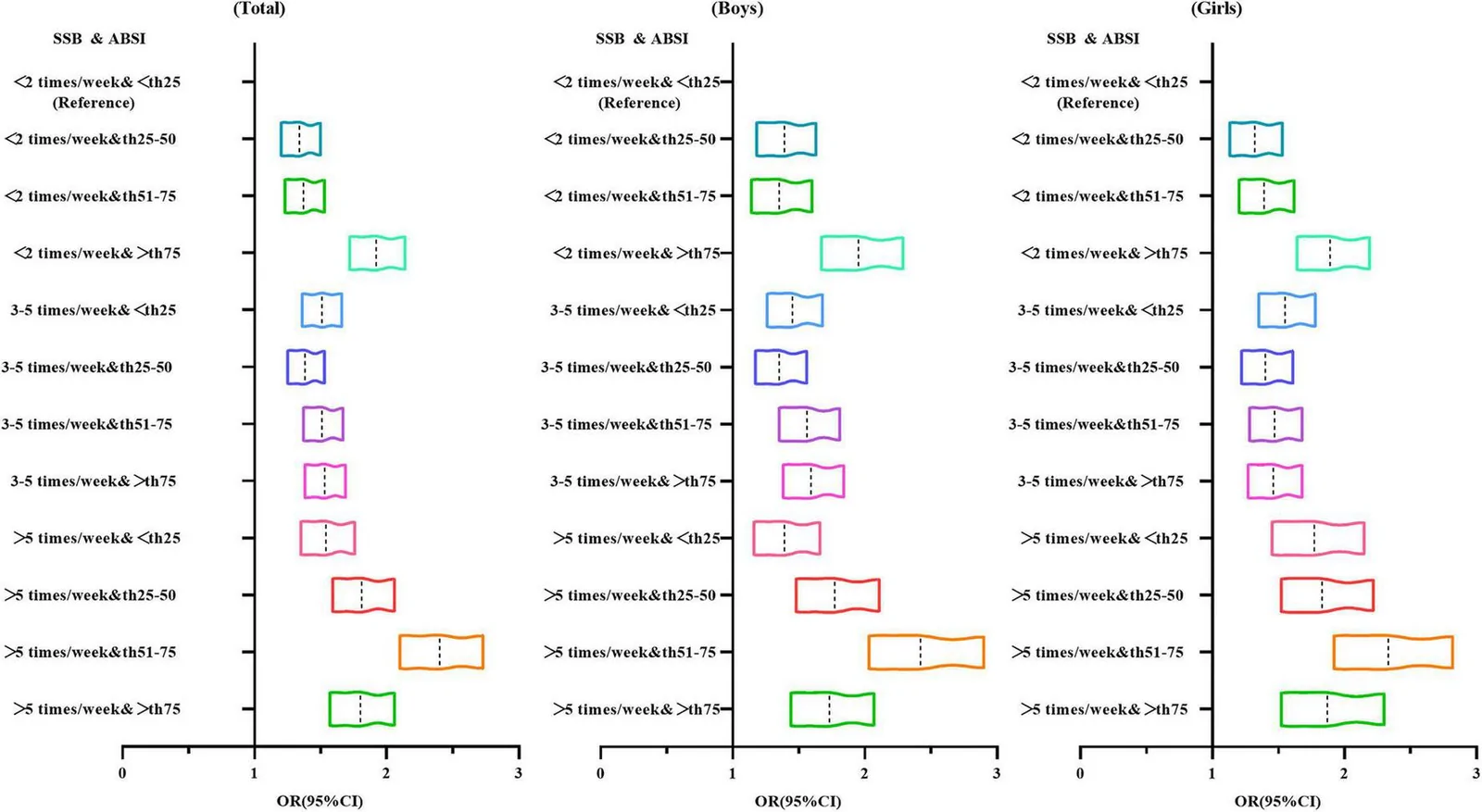
Trends in odds ratio values from binary logistic regression analysis of generalized linear models for SSB consumption, ABSI, and psychological symptoms among adolescents in rural China.

## 4 Discussion

The prevalence of psychological symptoms among adolescents has been steadily rising in recent years due to the combined influence of lifestyle, dietary habits, and other factors. This trend exerts significant negative impacts on academic performance, daily life during adolescence, and future physical and mental health in adulthood. The present study found a prevalence of psychological symptoms of 21.8% among rural Chinese adolescents, which is higher than the 20.3% reported by Zhang et al. in their nationwide survey of Chinese adolescents (10). At the same time, the findings of this study also exceed the results of Zhou et al.’s survey of Chinese adolescents (21.2%) (37). These findings suggest that the prevalence of psychological symptoms among adolescents in rural China is notably high, meriting considerable public health attention. Nevertheless, the evidence is not wholly uniform; a separate survey conducted among Chinese adolescents reported a prevalence of 26.17%, further underscoring the variability in estimates (9). The discrepancies in research findings stem from multiple factors. These differences are primarily associated with variations in the surveyed regions, participants’ ages, assessment questionnaires employed, and timing of the studies. The present study further reveals that among rural Chinese adolescents, males exhibit a higher prevalence of psychological symptoms than females, a result that stands in contrast to the findings of prior relevant research (38). The reason may stem from the influence of traditional Chinese cultural values, where males bear greater social and familial responsibilities. Concurrently, families place relatively higher educational expectations and demands on boys compared to girls. This results in adolescent males facing greater pressure and responsibilities, which may be one of the primary factors contributing to the findings of this study (39). On the other hand, when facing psychological issues, males often prefer to process and resolve them independently, whereas females tend to seek relief through confiding in peers. This factor may also be a significant reason why boys exhibit a higher prevalence of psychological symptoms than girls (40).

The study further demonstrated a positive association between SSB consumption and the prevalence of psychological symptoms, with increasing intake corresponding to elevated symptom rates. This result aligns with the conclusions of multiple studies. The reasons for this outcome are multifaceted. First, from the perspective of body composition, excessive SSB consumption has been associated with obesity, and adolescent obesity is associated with the presence of psychological symptoms. Research indicates that frequent SSB consumption is often accompanied by adverse body composition indicators, such as hyperlipidemia, high cholesterol, and mild inflammation, which themselves have been associated with increased odds of psychological symptoms in observational studies. For example, studies have found that adolescents in the highest quartile of weight-adjusted waist circumference index or body roundness index exhibit the highest risk of psychological symptoms (with odds ratios around 2.01) (41, 42). This suggests that sugar-sweetened beverages may negatively impact mental health by promoting central obesity or poor body composition, which in turn could contribute to body image concerns, metabolic inflammation, or insulin resistance. Secondly, from the perspective of dietary behavior, SSB consumption rarely occurs in isolation but is associated with overall unhealthy eating patterns. Research indicates that SSB consumption coexists with issues like fast food, fried foods, and ultra-processed food consumption. Concurrently, these unhealthy dietary behaviors are associated with the occurrence of psychological symptoms (43, 44). By contrast, a range of healthy dietary practices, such as vegetable and fruit intake, were found to be associated with a reduced likelihood of psychological symptoms (45, 46). This finding suggests that higher SSB consumption may indicate poorer overall dietary quality and nutritional imbalance. Deficiencies or excesses of certain nutrients have been hypothesized to alter the gut microbiota environment, which may influence brain function and emotional changes through the gut–brain axis. This may also contribute to psychological symptoms, although our study did not measure microbiome composition. Third, from an internal physiological mechanism perspective, SSB consumption is associated with high sugar intake and may cause significant fluctuations in blood glucose levels and disrupt neurotransmitters. Such physiological changes could, in turn, induce emotional fluctuations and thereby contribute to psychological symptoms (47). As our study did not include biomarkers of inflammation, insulin resistance, or gut microbiota, these mechanistic pathways remain speculative and require confirmation in dedicated biomarker studies. Additionally, excessive SSB consumption has been associated with addictive behaviors, which could form a vicious cycle with psychological distress. This may occur when negative emotions drive high sugar intake and the self-regulation of seeking sugary foods, ultimately triggering further negative emotional behaviors (48). Finally, as China’s economy continues to develop, living conditions in rural areas have improved, increasing the availability of sugar-sweetened beverages. This, in turn, has resulted in elevated SSB consumption, which has subsequently been associated with a higher prevalence of psychological symptoms.

A higher prevalence of psychological symptoms was also observed among rural Chinese adolescents with elevated ABSI scores, indicating a positive association between the two. First, ABSI effectively reflects central obesity, particularly abdominal adiposity (49). Previous research has linked abdominal fat accumulation to the development of negative emotions. Furthermore, studies suggest that abdominal obesity has been hypothesized to be associated with low-grade systemic inflammation, which in turn is closely associated with the presence of psychological symptoms (50). Secondly, an increase in body composition indicates the onset of obesity issues, leading to decreased satisfaction with one’s physical appearance among adolescents. This may result in bullying and teasing, negatively impacting their mental health (51). Research has found that a significant proportion of bullied adolescents are obese, which negatively impacts their mental health (52). Another meta-analysis encompassing 26 studies revealed that obese adolescents face a 46% higher risk of bullying compared to normal-weight children, while overweight children face a 24% higher risk. This risk appears to be more pronounced among boys (53). Finally, elevated ABSI levels are often associated with endocrine disorders in the body, and these disorders could affect brain health, contributing to the experience of negative emotional behaviors (54, 55). The findings of this study also indicate that the combined associations of SSB consumption and ABSI categories are associated with the odds of psychological symptoms. These findings further demonstrate that SSB consumption and ABSI are associated with psychological symptoms, both independently and in combination.

To the best of our knowledge, this study represents the first attempt to examine the independent and combined associations of SSB consumption and ABSI with psychological symptoms among rural Chinese adolescents, using a large multi-regional sample drawn from five provincial-level administrative regions. Nevertheless, several limitations should be acknowledged. First, the cross-sectional design precludes any causal inference regarding the observed relationships; future prospective cohort studies are warranted to establish causality. Second, the assessment of SSB intake, ABSI, and psychological symptoms relied on self-reported questionnaires, which may introduce measurement bias and recall error. More objective assessment tools should be employed in subsequent investigations to enhance analytical precision. Third, the determinants of adolescent psychological symptoms are multifactorial, yet our covariate set was restricted in scope. Important potential confounders – including physical activity, sedentary or screen time, smoking, alcohol use, family income, and caffeine intake – were not measured and therefore could not be adjusted for. Further studies incorporating these additional variables are needed to more accurately elucidate the associations examined here. Fourth, students were recruited through cluster sampling at the school level, and students within the same school tend to share dietary habits and family backgrounds. Because our regression models did not account for this clustering, the estimated confidence intervals may be somewhat narrower than those obtained from fully independent samples. Future analyses employing multilevel models or cluster-robust variance estimation are warranted.

## 5 Conclusion

Both independent and combined associations were observed between SSB consumption, ABSI, and psychological symptoms among adolescents in rural China. Adolescents with higher SSB consumption and higher ABSI exhibited a higher prevalence of psychological symptoms. Future interventions targeting psychological symptoms in rural adolescents may consider SSB consumption and ABSI as modifiable targets. Given the cross-sectional design, however, these associations should not be interpreted as causal.

## Data Availability

The raw data supporting the conclusions of this article will be made available by the authors, without undue reservation.
